# Telling right from right: the influence of handedness in the mental rotation of hands

**DOI:** 10.1186/s41235-020-00230-9

**Published:** 2020-06-03

**Authors:** You Cheng, Mary Hegarty, Elizabeth R. Chrastil

**Affiliations:** 1 University of California, Santa Barbara, Santa Barbara, CA USA; 2 University of California, Irvine, Irvine, CA USA

**Keywords:** Handedness, Mental rotation, World knowledge, Handedness strength, Motor imagery, Visual-proprioceptive integration

## Abstract

**Background:**

This study investigated the impact of handedness on a common spatial abilities task, the mental rotation task (MRT). The influence of a right-handed world was contrasted with people’s embodied experience with their own hands by testing both left- and right-handed people on an MRT of right- and left-hand stimuli. An additional consideration is the influence of matching the shape of the hand stimuli with the proprioception of one’s own hands. Two orthogonal hypothesis axes were crossed to yield four competing hypotheses. One axis contrasted (i) embodied experience versus (ii) world knowledge; the other axis contrasted (a) the match between the visual image of a hand on the screen and one’s own hand versus (b) the resemblance of the shape outline information from the hand stimuli with the proprioception of one’s own hands.

**Results:**

Among people with mixed handedness, right-handers performed more accurately for left-hand stimuli, while left-handers had a trend for higher accuracy for right-hand stimuli. For people with extreme handedness, right-handers outperformed left-handers. Regardless of group, there was no significant variation in performance for left-hand stimuli, with only right-hand stimuli producing significant variation.

**Conclusions:**

No hypothesis fully aligned with all the data. For left-hand stimuli, the consistent performance across groups does not provide support for embodied experience, while world knowledge might influence all groups similarly. Alternatively, the within-group variation for mixed-handed people supports embodied experience in the hand MRT, likely processed through visual-proprioceptive integration.

## Significance Statement

Ninety percent of human beings are right-handed. Accordingly, the world has been designed for right-handed use. But could spatial abilities be affected by knowledge of this right-handed world? If so, how does this world knowledge weigh against a person’s own embodied experience of their dominant hand when it comes to spatial thinking? Many psychologists tend to recruit only right-handed participants, but testing right-handed subjects alone cannot solve this puzzle, because their embodiment and world knowledge are indistinguishable. Since we all live in a right-handed world, we must test left-handed people to determine whether their spatial thinking diverges from that of right-handers. The mental rotation of hands shows a very different pattern of response from that of other mental rotation tasks (MRTs); the reason for this result has been assumed to be familiarity with hands. Thus, mental rotation of hands is a good candidate for testing whether embodiment or world knowledge influences spatial thinking. In the current study, we conducted a hand MRT in both left-handed and right-handed groups. Our findings demonstrate that embodied experience influences spatial thinking about right hands, which might account for the presence of world knowledge variability in MRT, while also suggesting that common external experience shapes performance in spatial thinking tasks. These findings demonstrate that investigations in spatial thinking tasks might overlook the nuances reflecting world knowledge versus embodied experience if researchers do not recruit left-handers.

## Background

### Hands are unique in mental rotation

The mental rotation of hands is a specific subtype of MRT in which the participants must determine whether two-dimensional hand pictures are the same (e.g., both are left hands or both are right hands) or different hands (a left hand and a right hand). In the mental rotation of hands, response time is much faster and more invariant to changes in orientation than for the mental rotation of other objects (Cooper, 1975; Cooper & Shepard, 1975; Folk & Luce, 1987; Shepard & Metzler, 1971; Stieff, 2007). The goal of the current study is to explore the cognitive mechanisms underlying this unusual effect using palm-up hand stimuli. Previous researchers have explained this unique effect in terms of people’s familiarity with hands (e.g., Parsons, 1987). However, two possible types of familiarity could exist for right or left hands: embodied experience and world knowledge. These two types of familiarity, by extension, lead to two different theories that could help explain the mental rotation of hands.

#### Embodied experience theory

Hands, as parts of the human body, provide us with embodied experience through our interaction with the world. The hand that is used more often in daily life—the dominant hand—typically provides more embodied experience. Therefore, people are likely to have greater embodied experience with their dominant hand than with their nondominant hand. Consistent with this theory, one study showed that people who had lost their dominant hand responded more slowly and less accurately in a hand MRT than people who had lost their nondominant hand (Nico, Daprati, Rigal, Parsons, & Sirigu, 2004). This finding suggests the importance of embodied experience, especially from dominant hands, in the mental rotation of hands.

#### World knowledge theory

Approximately 90% of human beings are right-handed, defined either by skill or by preference in spite of culture or ethnicity (Coren & Porac, 1977; Previc, 1991). Almost all tools and facilities (e.g., scissors, notebooks, spiral staircases) in daily life are designed for right-handed people. In other words, we live in a right-handed world and have more experience seeing people use their right hands (e.g., for writing) than their left hands. Therefore, the possibility exists that everyone is more familiar with right hands than left hands, regardless of their own handedness. In support of this theory, a study of split-brain patients revealed that the right hand has an advantage in representing acquired tool use, regardless of whether the person is right- or left-handed (Frey, Funnell, Gerry, & Gazzaniga, 2005). This result suggests that experience in a right-handed world could affect the abilities of even left-handers. Perhaps more direct evidence comes from a study in which researchers put left-handed mice in a right-handed world (their food was put in the right corner of an environment such that it was much easier to access with the right paw), and some of the left-handed mice changed to be right-handed (Collins, 1975). This finding indicates that the design of the world for right-handers is so powerful that it can potentially overcome natural proclivities. Other research has described how left-handers struggle living in this right-handed world (e.g., Masud & Ajmal, 2012; Suitner, Maass, Bettinsoli, Carraro, & Kumar, 2017; Zaghloul, Saquib, Al-Mazrou, & Saquib, 2018).

The present study tested the extent to which people’s spatial thinking is influenced by world knowledge and embodied experience. A good way to test these two theories is by contrasting performance between left-handers and right-handers. One theory is that mental rotation of hands is supported by world knowledge, which provides more familiarity with right hands than with left hands for all people. Under this theory, we predict that all people will perform better on right-hand stimuli than on left-hand stimuli, independent of their handedness. In contrast, if the mental rotation of hands is supported by embodied experience, an advantage for the dominant hand is expected. Thus, left-handers are predicted to have better performance for left-hand stimuli, and right-handers are predicted to have better performance for right-hand stimuli.

Two primary tasks have been developed to study the mental rotation of hands. One task is the hand laterality task (HLT), meaning the person must determine whether a single hand shown on the screen is a left hand or a right hand. This task is simple but is prone to verbal labeling errors. The second type of task is a modified Shepard and Metzler task (SMT) in which subjects are presented with two hands simultaneously, one on each side of the screen. The task is to decide whether the two hands are the same (both left hands or both right hands) or different (one is a left hand; one is a right hand). The HLT is used more often to study motor behavior (e.g., Parsons, 1994), while the hand version of the SMT is more commonly tested together with SMT of other stimuli (e.g., tools, letters, cubes) to illustrate the striking unique reaction time pattern in the mental rotation of hands. In addition, the laterality task is classified as an egocentric perspective transformation because spatial information is formed with respect to oneself, whereas the SMT is an object-based spatial transformation because spatial information is formed independent of the observer’s view (Zacks, Mires, Tversky, & Hazeltine, 2002). Although an egocentric perspective could be advantageous, since participants can imagine rotating their own hand to complete the task, there are also a number of biomechanical limitations to this perspective (Parsons, 1987), described in detail below. Accordingly, we used the SMT for this study.

### Handedness

#### Strength of handedness

In addition to studying the direction of handedness (i.e., left or right), another thread of research focuses on the strength of handedness. The strength of handedness varies from mixed (inconsistent hand preference for activities) to extreme (very consistent in using either the left or the right hand). There is some evidence that extreme-handed individuals—whether right- or left-handed—have less cognitive flexibility than mixed-handed individuals (Badzakova-Trajkov, Häberling, & Corballis, 2011; Barnett & Corballis, 2002; Nicholls, Orr, & Lindell, 2005). In contrast, mixed-handed individuals perform better on memory tasks that require hemispheric interaction (e.g., paired associate recall) (Lyle, McCabe, & Roediger, 2008; Lyle & Orsborn, 2011; Propper, Christman, & Phaneuf, 2005). Mixed-handed individuals also have better memory for the frequency of using one hand or the other in everyday unimanual tasks (e.g., brushing one’s teeth) (Edlin, Carris, & Lyle, 2013). Because extreme-handers have more embodied experience with their dominant hands than mixed-handers, we expect subjects’ performance for right-hand stimuli will increase from extreme left-handers having the worst performance, to mixed left-handers, to mixed right-handers, to extreme right-handers having the best performance; performance for left-hand stimuli is expected to show the reverse pattern among handedness groups. Thus, we included people with both mixed and extreme handedness in the sample (although due to their relative rarity, the sample size of extreme left-handers is fairly small), aiming to take a closer look at the influence of handedness strength.

#### Handedness effects on the mental rotation of hands

To our knowledge, only one previous study has tested the influence of handedness on the mental rotation of hands. The researchers used six hand gestures in the HLT, including one palm-up and five palm-down gestures. They found a reaction time advantage for right-hand stimuli in right-handers, but they also showed a speed–accuracy trade-off (Ní Choisdealbha, Brady, & Maguinness, 2011). No difference in performance between left and right-hand stimuli was found in left-handers. These results indicate that left-handers and right-handers might have different mechanisms for responding to left-hand stimuli and right-hand stimuli in the mental rotation of hands. They also found that reaction time for all five palm-down gestures showed a standard pattern across rotation angles, while the palm-up gesture peaked at a larger rotation angle, indicating that the palm-up gesture might be treated differently from other gestures.

Prior to Ní Choisdealbha’s work, Sekiyama (1982) studied mental rotation of hands with five different hand gestures (three in a palm-up position, two in a palm-down position) in right-handed people by using a hand laterality paradigm. Like Ní Choisdealbha’s research, one big difference was found between palm-up left-hand stimuli and palm-down right-hand stimuli. Specifically, the reaction time pattern of the degree of *clockwise* rotations for the palm-up left-hand stimuli was similar to that of the *counterclockwise* rotations for the palm-down right-hand stimuli, indicating a “wrong-hand” effect that will be explained in more detail later. We next turn to how sensory and motor systems could explain these effects.

### Sensorimotor theories of the mental rotation of hands

#### Motor simulation theory

The traditional view of the mental rotation of hands is based on motor simulation theory. Under this theory, the motor system that guides the intended action is automatically activated during the mental rotation of hands, which could cause a feeling of moving (Parsons, 1987, 1994; Parsons, Gabrieli, Phelps, & Gazzaniga, 1998). This theory suggests an alignment between the spatial representation of the subject’s own hand and the image of a hand on the screen: An image of a left hand, for example, will always align with the subject’s left hand (regardless of whether it is palm up or palm down) because the motor system requires a consistent internal representation of body position.

In the 1980s, Lawrence M. Parsons carried out a series of studies to test the motor simulation theory for the mental rotation of hands by using HLTs. In his tasks, participants viewed hand stimuli from different perspectives, with the orientation varying from the normal physical range of motion to an awkward range that is difficult to produce biomechanically (Parsons, 1987). For example, a palm-down left hand turned in a counterclockwise direction would be considered an “awkward” orientation, whereas a palm-down left hand turned in a clockwise direction would be considered “normal” range. Parsons found that across all hand views, awkward orientations took longer than normal orientations for both right and left hands. When a palm-down hand stimulus was viewed, reaction time increased slightly with each increasing angle of orientation for both normal and awkward orientations. When a palm-up hand stimulus was viewed, however, Parsons found a flat reaction time pattern for normal orientations and a pattern with a peak for awkward orientations. These results indicate that mental rotation of palm-up hands is relatively more invariant to changes in orientation than mental rotation of palm-down hands.

It is possible that handedness could contribute to some of those findings. However, all the participants recruited in Parsons’ study (1987) were right-handed. Those right-handed participants were slower overall in responding to left-hand stimuli than to right-hand stimuli for both the palm-up and the palm-down gestures, although this effect was more robust for palm-down hands. This finding suggests that right-handers have an advantage in responding to right-hand stimuli compared with left-hand stimuli, supporting the embodied experience hypothesis.

In order to explore the influence of the HLT itself, Parsons (1987) asked subjects to complete the same experiment by imagining transforming their own hands to the position of the presented hand stimuli. Subjects only needed to verbally report “now” to indicate that they had completed the mental spatial transformation. In normal orientations, reaction time was faster for right hands than for left hands when the stimuli were palm-down hands, but this advantage switched to be faster for left hands than for right hands when the stimuli were palm-up hands. This result suggests some kind of confusion about the shape of the hand, or a “wrong-hand effect.” As these studies specifically required participants to imagine transforming their own hands, the similar results for both studies suggest that the preferred strategy in a HLT is to imagine moving one’s hand to simulate the orientation of the stimulus.

#### Visual-proprioceptive integration theory and the wrong-hand effect

Viswanathan, Fritz, and Grafton (2012) challenged the conventional view of motor simulation processes underlying the mental rotation of hands by proposing a visual-proprioceptive integration theory (Grafton & Viswanathan, 2014; Viswanathan et al., 2012). Under the visual-proprioceptive integration theory, information from different sensory modalities is integrated to enable a coherent experience of an object. In the case of the mental rotation of hands, the hand stimuli on the screen and the subject’s own hand share a spatial feature (e.g., outline shape or digit ratio). Visual-proprioceptive integration is the processing of that shared spatial information. In this case, it is the multisensory integration of the visual input of the spatial configuration (the outline or “shape”) of the image of a hand on the screen and the proprioceptive input (information about where each body part is) of the response hand. Note that in this theory, visual details indicating whether the hand is palm up or palm down are ignored, and only the outline shape of the hand is considered. In their task, the subject’s response hands—both left and right hands—were in a palm-down position to make their responses on the keyboard. This setup created a shape match: The shape of a right hand in a *palm-up* gesture on the screen matches the shape of the *palm-down* left hand of the subject making the response, and vice versa for a left-hand palm-up.

The researchers found that people’s hand laterality judgments can be easily manipulated by the sequence of perceptual processing of the shape and view of a hand (Viswanathan et al., 2012). The stimuli presentation was manipulated so that participants preferentially processed either shape information or view information—the information about whether the person is looking at the palm or back of the hand. The hand stimulus consisted of a visual outline of a hand (a black silhouette), with a colored dot as the only indication of whether the hand was palm up or palm down. When the researchers cued the trials such that view information was preferentially processed, a left palm-up gesture on the screen was recognized as a left hand and vice versa for a right palm-up gesture. When the shape information was cued to be preferentially processed; however, a left palm-up gesture on the screen was recognized as a right hand and vice versa for a right palm-up gesture, suggesting that people were biased toward processing an ambiguous shape as the back of the hand. This “wrong-hand effect” could be due to the premature binding of the observer’s felt hand, which was palm down, with the ambiguous hand on the screen.

However, it is unknown whether shape information is processed separately when both shape and details showing whether it is the palm or the back of the hand are presented simultaneously. In order to answer this question, in the present study, we tested stimuli with details clearly showing that it is the palm of the hand.

### Hypotheses and predictions

The goal of the present study was to explore the cognitive mechanisms underlying the mental rotation of hands. In this experiment, left-handed and right-handed subjects were recruited to complete a modified SMT with hand stimuli. We started with the goal of purely exploring the influence of world knowledge and embodied experience, but we also needed to address the additional contrasting hypotheses regarding the information-processing mechanisms of hand mental rotation (motor imagery and visual-proprioceptive integration). Therefore, we crossed two orthogonal hypothesis axes to yield four competing hypotheses. One axis of the hypothesis space contrasted (i) world knowledge of a right-handed world versus (ii) embodied experience with one’s own hands; the other hypothesis axis contrasted (a) motor imagery (i.e., motor simulation) versus (b) visual-proprioceptive integration based on shape information alone (i.e., the wrong-hand effect). A brief overview of the predictions of each of the theories is stated below.

i. **World knowledge.** Because left-handers and right-handers share the same knowledge of a right-handed world, this theory predicts better performance for right-hand stimuli than for left-hand stimuli for the mental rotation of hands for all individuals. If all subjects respond faster or more accurately to right-hand stimuli, then this hypothesis would be supported.
ii. **Embodied experience.** An alternative theory is that people respond better to a hand stimulus that matches their dominant hand. If left-handers respond faster or more accurately for left-hand stimuli and right-handers respond faster or more accurately for right-hand stimuli, then the embodied experience hypothesis would be supported.

- **Motor imagery.** Under the motor imagery theory (i.e., motor simulation theory), the match between one’s own hand and the hand seen on screen is based on the hand’s details (e.g., shape, visual details). The view of a right hand in any orientation automatically activates a motor representation of a person’s right hand, and the view of a left hand activates the motor representation of a person’s left hand. While this hypothesis cannot on its own indicate how handedness influences performance, it makes predictions in combination with world knowledge or embodied experience. Specifically, when combined with either world knowledge or embodied experience it predicts better performance by right-handers for right-hand stimuli. However, for left-handers, it predicts better performance for right hands under world knowledge and better performance for left hands under embodied experience.
- **Visual-proprioceptive integration.** Under this theory, a “wrong-hand effect” will be expected, whereby the match of spatial configuration (“shape”) of the hand stimuli on the screen and the proprioceptive information from the hand making the response is preferentially processed. We tested only palm-up stimuli in our experiment, and the hand making the response in this task was in a palm-down position on the keyboard. Thus, this theory predicts that right-handed subjects will perform better for left-hand stimuli than for right-hand stimuli, regardless of embodiment or world knowledge, which is contrary to the prediction of motor imagery theory. Left-handed people will perform better for right hands if combined with embodiment, and better for left hands if combined with world knowledge.

**Fig. 1:**
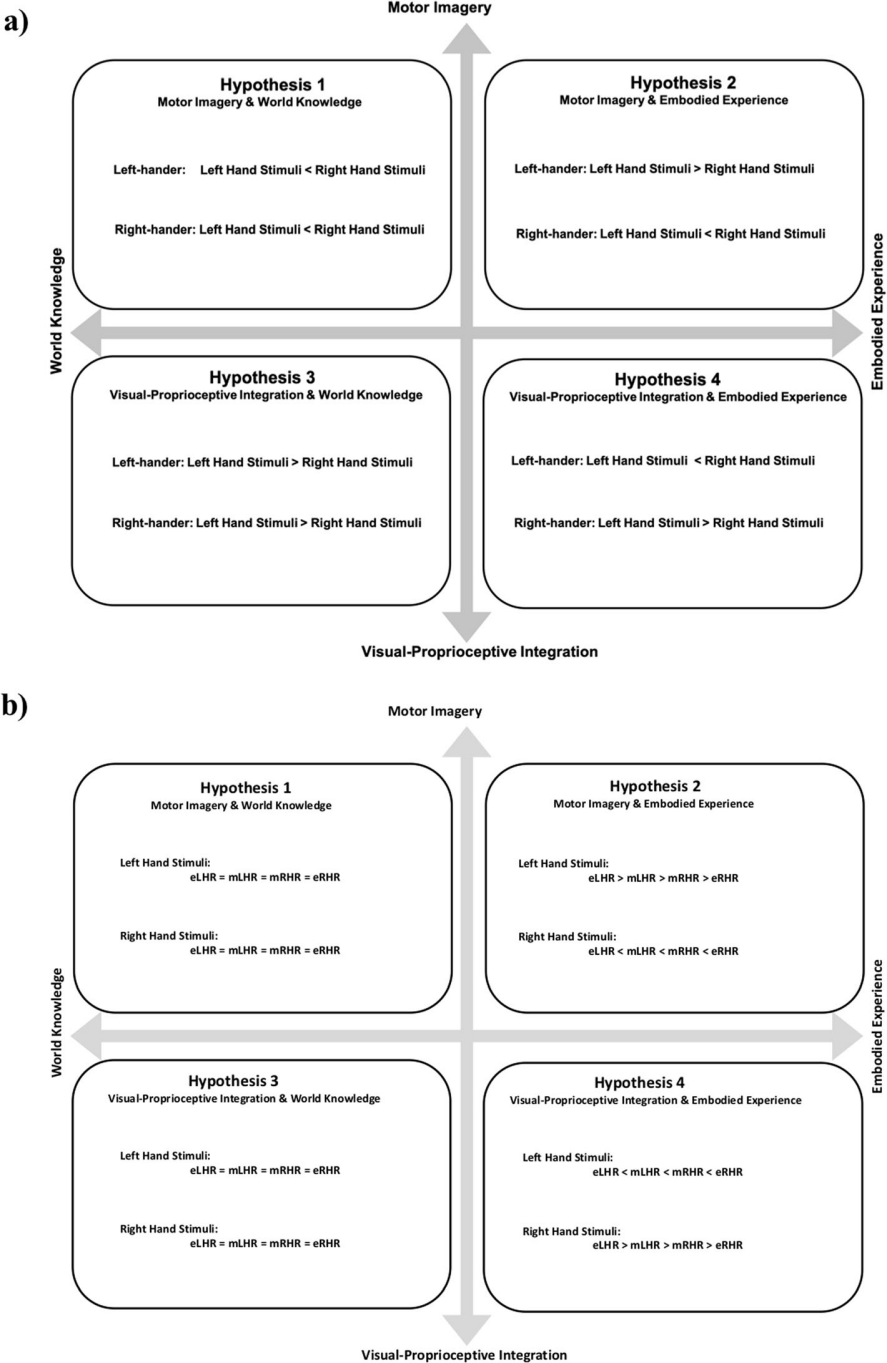
Hypotheses and predictions. **a** Predictions based on the subject’s handedness, indicating which stimulus will have greater performance for each group. **b** Predictions based on hand stimuli tested, indicating which handedness group will perform best for left- and right-hand stimuli. *eLHR* Extreme left-handers, *eRHR* Extreme right-handers, *mLHR* Mixed left-handers,, *mRHR* Mixed right-handers

These two sets of theories represent orthogonal features in the mental rotation of hands. In order to fully test the interaction of these two sets of theories, we crossed these two pairs of theories to yield four specific hypotheses. Figure 1 illustrates these four hypotheses, by showing predictions both based on the handedness of the individual (Fig. 1a) and based on the hand stimulus (Fig. 1b).

### Hypothesis 1: motor imagery and world knowledge

Prediction: First, based on motor imagery theory, a left palm-up hand stimulus will be recognized as a left hand, and a right palm-up hand stimulus will be recognized as a right hand. Second, based on world knowledge theory, everyone will be more familiar with right hands than with left hands. Therefore, all subjects’ performance for right-hand stimuli will be better than for left-hand stimuli. No differences are expected between mixed- and extreme-handed people in this hypothesis.

### Hypothesis 2: motor imagery and embodied experience

Prediction: First, based on motor imagery theory, a left palm-up hand stimulus will be recognized as a left hand and vice versa for right hands. Second, based on embodied experience theory, people will perform better on stimuli that match their dominant hands than on stimuli that match their nondominant hands. Therefore, left-handers will perform better for left-hand stimuli, and right-handers will perform better for right-hand stimuli. Third, extreme-handers will have more embodied experience with their dominant hands than mixed-handers. Thus, extreme-handers will perform better on stimuli that match their dominant hands than mixed-handers and will perform worse on stimuli that match their nondominant hands than mixed-handers.

### Hypothesis 3: visual-proprioceptive integration and world knowledge

Prediction: First, based on visual-proprioceptive integration theory, a left palm-up hand stimulus will be recognized as a right hand and vice versa for right hands. Second, based on world knowledge theory, everyone will be more familiar with right hands than with left hands. Therefore, under this hypothesis, all subjects’ performance for left-hand stimuli will be better than for right-hand stimuli. No differences are expected between mixed- and extreme-handed people in this hypothesis.

### Hypothesis 4: visual-proprioceptive integration and embodied experience

Prediction: First, based on visual-proprioceptive integration theory, a left palm-up hand stimulus will be recognized as a right hand and vice versa for right hands. Second, based on embodied experience theory, people will perform better on stimuli that match their dominant hands than on stimuli that match their nondominant hands. Therefore, for left-handers, performance for right-hand stimuli will be better than for left-hand stimuli. For right-handers, performance for left-hand stimuli will be better than for right-hand stimuli. Third, extreme-handers will have more embodied experience with their dominant hands than mixed-handers. Thus, extreme-handers will perform better than mixed-handers on palm-up stimuli that match the shape of their dominant hands and will perform worse on palm-up stimuli that match the shape of their nondominant hands. To distinguish between these four hypotheses, we tested both left-handed and right-handed subjects and incorporated left- and right-hand stimuli on the screen.

### Other possible factors

Besides handedness direction, some other factors might influence subjects’ performance. Although we tried to control the influence of these factors in our experimental design, it is still possible that they could influence the outcomes. Thus, we will still consider them at a later point in our data analysis in order to have a more thorough understanding of the results. Here we introduce some of the main additional possible factors and how we tried to control them.

#### Hand gestures

Most previous studies on the mental rotation of hands only used one gesture as a stimulus, usually an open-palm gesture, but these studies also tended to have a ceiling effect (e.g., de Lange, Helmich, & Toni, 2006; Parsons, 1987; Zapparoli et al., 2014). The use of a single gesture could be one factor leading to this ceiling effect, so we added a hand in a pointer gesture to increase the difficulty of the test. To make the task even more challenging, we also included a condition in which the two hand stimuli were different gestures (one pointer, one palm). Because of these modifications, we expected that response accuracy could become another performance indicator in the study, in addition to reaction time.

#### Response pattern

Here, response pattern refers to which hand pressed the “same” response and which hand pressed the “different” response. For this study, there were two response patterns: left hand pressed “same” and right hand pressed “different”, or left hand pressed “different” and right hand pressed “same.” We counterbalanced this factor by randomly assigning half of the subjects in each handedness group to complete the task with each response pattern; however, we did not analyze this factor specifically.

#### Strategy

Two primary strategies could be used in this hand MRT: mental rotation and thumb strategies. Mental rotation means solving the problem purely by mentally rotating one hand stimulus to match the other one. The thumb strategy is a trick, comparing whether the thumb is on the same side of each hand stimulus. For example, if there are two left hands on the screen, both thumbs are on the left side of each hand, regardless of their rotation angles, because all hand stimuli in this experiment were palm-up. As mentioned above, extreme-handers tend to be less cognitively flexible than mixed-handers. Thus, it is possible that mixed-handers would have a higher frequency of applying the thumb strategy, while extreme-handers would tend to rely on mental rotation. Therefore, we analyzed strategy related both to handedness direction and to handedness strength.

## Methods

### Participants

Participants consisted of 69 (41 females) University of California, Santa Barbara (UCSB), undergraduates who participated in return for course credit. We conducted a *post hoc* power analysis of our sample size using G*Power software (http://www.gpower.hhu.de/) (Erdfelder, Faul, & Buchner, 1996). Although our design was with a three-way analysis of variance (ANOVA), current G*Power power analyses are limited to two-way ANOVA. Using that design, we estimated that with an alpha = 0.05, *n* = 66 (after dropouts), and Cohen’s *f* measurement of effect size = 0.25, the resulting power for between-group comparisons was 0.63. We made a special call for left-handed subjects in our subject recruitment system so that we could recruit the same number of left-handers and right-handers in our study. Participants were discarded from data analysis for using their own hands to simulate hand stimuli (*n* = 2) or having a high proportion of reaction time outliers (*n* = 1). Ages of the remaining 66 participants ranged from 18 to 24 years (mean, 19.70 years; two participants did not report their age). The direction and strength of each person’s handedness was tested using the Edinburgh Handedness Inventory (EHI; see Appendix) (Oldfield, 1971). The final analysis included 33 left-handers and 33 right-handers, comprising 23 mixed left-handers (14 females), 10 extreme left-handers (7 females), 16 mixed right-handers (8 females), and 17 extreme right-handers (10 females). All participants signed an informed consent form in agreement with the UCSB Institutional Review Board requirements and in accordance with the principles of the Declaration of Helsinki.

### Materials

**Fig. 2:**
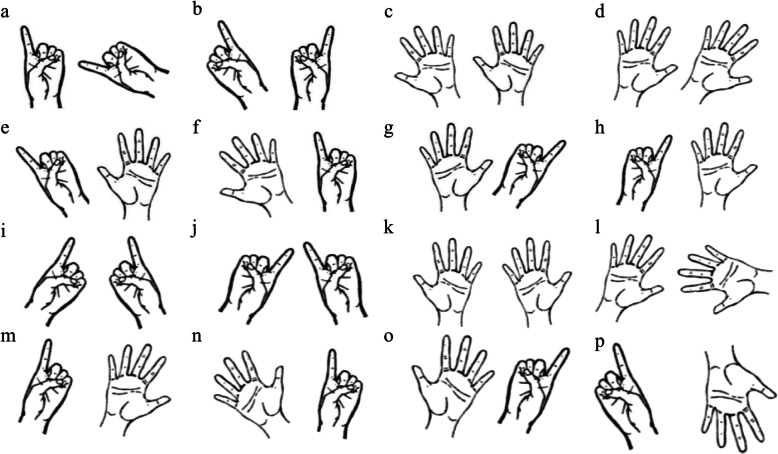
All hand stimuli combinations. The same hand condition (**a**–**h**) includes ten pairs each, and the different hand condition includes (**i**–**p**) includes ten pairs each

We used a modified SMT with hand stimuli, which were adapted from a previous study (Sperry, 1968). All stimuli were palm-side up but could have the palm either open or closed in a pointing gesture (see Fig. 2). All images (500 × 500 pixels for each image) were displayed to the participants on a 15-in. computer monitor (display resolution at 1920 × 1080 pixels) using E-prime 2.0 software (Schneider, Eschman, & Zuccolotto, 2012). For each trial, one hand was displayed on the left side of the screen, and the other hand was displayed on the right side of the screen.

### Design

A 2 (handedness direction: left-handed, right-handed) × 2 (handedness strength: extreme, mixed) design was used for the between-subjects variables. The within-subjects portion of the design was 2 (stimulus condition: the two stimuli showed the same hand, or they showed different hands) × 2 (gesture type: same gesture, different gesture) × 2 (subtypes of each gesture type: palm-palm and pointer-pointer for the same gesture combination, palm-pointer [palm on the left] and pointer-palm [palm on the right] for the different gesture combination) × 10 (the angular disparity between the two hands: 10 magnitudes ranging from 0 to 180 degrees in 20-degree steps; however, not all subtypes of gesture combinations were included in all of the possible angular disparities). The position of the hands was counterbalanced such that a left hand could appear equally often on the left and right sides of the screen. Therefore, there were 160 trials in total (2 same/different hand × 2 same/different gesture × 2 subtypes of each gesture type × 10 angular disparities × 2 counterbalancing positions), although the 10 angular disparities were not evenly distributed across all conditions (Fig. 2). These 160 trials were randomly separated into two blocks with a short break between them. All stimuli were presented in random order for all participants.

### Procedure

Subjects first were greeted in the lab, given information about the study, and given consent forms to sign. They then completed the EHI (see Appendix). The EHI questionnaire contains ten items of daily behaviors (e.g., writing). Subjects were asked to fill in blanks with “+” or “++,” indicating the frequency of using their left hand or right hand for those behaviors in daily life, with “++” indicating greater frequency.

**Fig. 3:**
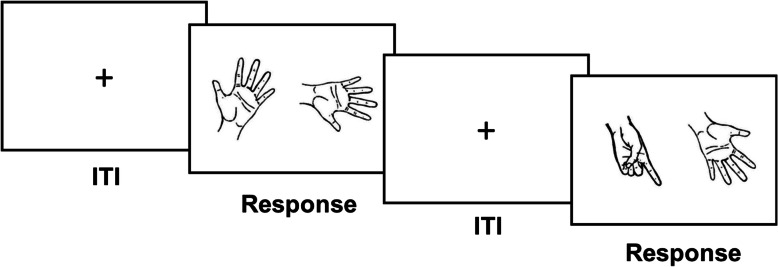
The flow of two trials. The intertrial interval (ITI) is 1000 ms. During the response, the task of the participant is to decide whether the two hand stimuli are the same hand or different hands

Next, they were given instructions and performed the mental rotation of hands task. Subjects sat approximately 50 cm in front of the computer screen. They were first presented with instructions to understand the task, then started with four practice trials (stimuli were different from experimental trials) before beginning the formal experiment. Each trial started with a fixation cross for 1000 ms as the intertrial interval. Then, two hand stimuli were presented simultaneously on the left and right sides of the screen. Subjects were asked to judge whether the two hand stimuli were the same hands or different hands (Fig. 3). They used one hand to press a key to indicate that the stimuli were the same hands and used the other hand to press another key to indicate that the stimuli were different hands; whether the hand used to respond to the “same” trials was their dominant or nondominant hand was counterbalanced across subjects. Subjects were instructed to respond as quickly and accurately as possible. The 1000-ms fixation cross for the next trial started automatically as soon as subjects pressed a response key. Accuracy and reaction time for each trial were recorded.

We considered strategy to be an additional factor that could potentially influence subjects’ performance. At the end of the experiment, participants were asked to verbally report their strategy in completing the tasks, which the experimenter wrote down. Two primary strategies were used in this hand MRT: mental rotation and thumb strategies, which are described in the Background section above.

### Data analysis

We evaluated subjects’ handedness direction and strength based on their EHI scores. The laterality quotient (LQ) was calculated on the basis of the sum of the left “+” marks (L) and the sum of the right “+” marks (R):

$$ LQ=\left(R-L\right)/\left(R+L\right)\times 100 $$

**Fig. 4:**
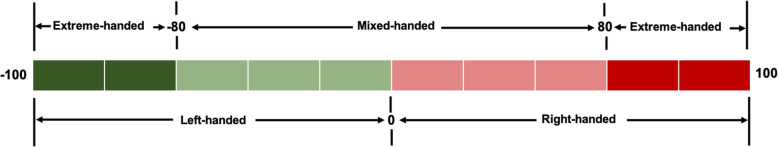
Handedness categorization measured by the Edinburgh Handedness Inventory. For handedness direction, if the laterality quotient (LQ) score was within the range of − 1 to − 100, then the subject was considered left-handed. If the LQ score was within the range of + 1 to + 100, then subject was considered right-handed. For handedness strength, if the LQ score was between − 80 and + 80, then the handedness strength was mixed. If the LQ score fell in ranges of either − 100 to − 80 or + 80 to + 100, then the handedness strength was assigned as extreme

We measured each subject’s handedness direction and handedness strength on the basis of the criteria used in previous studies (Christman & Butler, 2011; Hardie & Wright, 2014; Lyle & Orsborn, 2011; Smit, Kooistra, van der Ham, & Dijkerman, 2017; Westfall, Jasper, & Christman, 2012). For handedness *direction*, if the LQ score was within the range of − 1 to − 100, then the subject was considered left-handed. If the LQ score was within the range of + 1 to + 100, then subject was considered right-handed. For handedness *strength*, if the LQ score was between − 80 to + 80, then the handedness strength was mixed. If the LQ score fell in ranges of either − 100 to − 80 or + 80 to + 100, then the handedness strength was assigned as extreme (see Fig. 4).

For the task data, we first removed outliers that were below or above 2 standard deviations of the mean of each subject’s reaction time; approximately 1.78% of trials were removed. Then, we calculated the accuracy of the remaining trials. The reaction times for each condition were calculated only on the basis of correct trials. Because congruency cannot be defined in “different” trials, previous studies usually analyzed only “same” trials (e.g., Shepard & Metzler, 1971). To examine the effect of the laterality of the hand stimuli (left hands vs. right hands), we had to analyze pairs that displayed either both left or both right hands. Mismatches displayed both left and right hands on the screen; because our hypotheses are specific to either right-hand or left-hand stimuli, mismatches could not distinguish between our hypotheses. Therefore, for the subsequent analysis, we examined only trials on which the two hands on the screen were the same, either both right hands or both left hands.

Prior to the formal data analyses, we conducted overall analyses of the full dataset to gain knowledge of the dataset under each condition more generally. For the data analysis, we conducted a 2 (handedness direction: left-handed, right-handed) × 2 (handedness strength: extreme, mixed) × 2 (hand stimuli tested: left hands, right hands) mixed ANOVA only for same-hand trials. “Hand stimuli tested” was a within-subject factor, while “handedness direction” and “handedness strength” were between-subject factors. In the data analysis, we did not initially consider gesture combination as a factor, because the inclusion of different gestures was mainly designed to increase task difficulty and was not a primary factor of interest. We did not separately consider response pattern (which hand was used to press “same” or “different” key), because the counterbalanced design minimized the influence of the response hand. We also did not have sufficient power to conduct analysis on the angular disparity. RStudio (https://rstudio.com/) was used for all data analyses.

## Results

### Overall analyses

We first examined the overall effects of both between-subject and within-subject factors to gain knowledge of the dataset under broad conditions of interest (e.g., male vs. female subjects’ trials). This was a general assessment of the data distribution in all related factors rather than in-depth data analysis to answer the research questions, which will be explicitly discussed in the next section. For the between-subjects effects, we conducted two-sample *t* tests on the accuracy and reaction time of all trials (both “same” and “different” trials combined) on the primary between-subjects variables of handedness direction, handedness strength, and response pattern (whether the left hand pressed “same” and the right hand pressed “different” or vice versa). Because sex differences have previously been found in mental rotation studies (e.g., Voyer, Voyer, & Bryden, 1995), we also conducted a two-sample *t* test on sex. We found no differences in overall accuracy or reaction time between left-handers and right-handers, between extreme-handed individuals and mixed-handed individuals, between the left hand pressing “same”/right hand pressing “different,” and the right hand pressing “same”/left hand pressing “different” response patterns, or between males and females (all *p* > .1).

For the within-subject effects, we conducted paired *t* tests on the accuracy and reaction time of all trials on same versus different hand stimuli and same versus different gesture of the hand stimuli. We found no difference in accuracy between same-hand trials (*M* = 89% ± 1% *S.E.*) and different hand trials (*M* = 89% ± 1%) [*t*(65) = − 0.43; *p* = .67, ns], but the reaction time for same hand trials (*M* = 3058 ± 137 *S.E.* ms) was significantly faster than for different hand trials (*M* = 3463 ± 159 ms) [*t*(65) = − 6.28; *p* < .001; Cohen’s *d* = − 0.773]. As for the gesture of the hand stimuli, there was no difference in accuracy [*t*(65) = − 0.24; *p* = .812, ns] between same stimuli gestures (*M* = 89% ± 1%) and different stimuli gestures (*M* = 89% ± 1%), but reaction time was significantly shorter [*t*(65) = − 5.16; *p* < .001; *d* = − 0.635] for same stimuli gestures (*M* = 3087 ± 138 ms) than for different stimuli gestures (*M* = 3440 ± 156 ms).

### Effects of handedness direction, handedness strength, and hand stimuli tested

#### Same-hand trials

We first conducted a 2 (handedness direction: left-handed, right-handed) × 2 (handedness strength: extreme, mixed) × 2 (hand stimuli tested: left hands, right hands) ANOVA on trials in which both stimuli were of the same hand (see Table 1). For accuracy, there were no significant main effects or interactions (all *p* > .1). There was a marginally significant interaction between handedness direction and handedness strength [*F*(1,62) = 3.28; *p* = .07; *η*_*p*_^*2*^ = 0.05], but since this was only a marginal trend, we did not examine it further.

**Table 1:** Descriptive statistics for accuracy of same-hand trials

Condition	Left-handed				Right-handed			
	Mixed		Extreme		Mixed		Extreme	
	*M*	*SD*	*M*	*SD*	*M*	*SD*	*M*	*SD*
Left-hand stimuli	86%	11%	85%	14%	89%	17%	91%	8%
Right-hand stimuli	90%	11%	83%	13%	84%	23%	93%	7%

*Note*: *M* Mean; *SD* Standard deviation

For reaction time (see Table 2), there were again no significant effects, but we found a marginal main effect of hand stimuli tested [*F*(1,62) = 3.33; *p* = .07; *η*_*p*_^*2*^ = 0.05], such that the reaction time for right-hand stimuli (*M* = 3024 ± 100 ms) was somewhat faster than for left-hand stimuli (*M* = 3150 ± 118 ms). We found a marginally significant three-way interaction among handedness, hand tested, and strength in reaction time [*F*(1,62) = 2.98; *p* = .09; *η*_*p*_^*2*^ = 0.05, *ns*]. Since this was only a marginal trend, we did not examine it in more depth. Other than these two results, there were no significant main effects or interactions (all *p* > .1). Therefore, overall, there were no effects for same hand trials, except for a marginal tendency to respond faster for right-hand stimuli.

**Table 2:** Descriptive statistics for reaction time of same-hand trials

Condition	Left-handed				Right-handed			
	Mixed		Extreme		Mixed		Extreme	
	*M*	*SD*	*M*	*SD*	*M*	*SD*	*M*	*SD*
Left-hand stimuli	3125	1143	3222	884	3245	1904	3052	1298
Right-hand stimuli	3135	1178	3116	781	2802	1361	3026	1091

*Note*: *M* Mean; *SD* Standard deviation. Reaction time is in milliseconds (ms)

#### Same hand/same gesture

As half of the same-hand trials were different gestures (one pointer, one palm), which has not been tested in previous studies, there might be a difference in performance between same gesture (both palms or both pointers) and different gestures (one palm and one pointer). This difference between gestures could yield noise that might overshadow the main effect. Therefore, for consistency with previous studies (e.g., de Lange et al., 2006; Parsons, 1987; Zapparoli et al., 2014), we next conducted a detailed analysis of trials in which the gesture was the same for both hand stimuli.

**Fig. 5:**
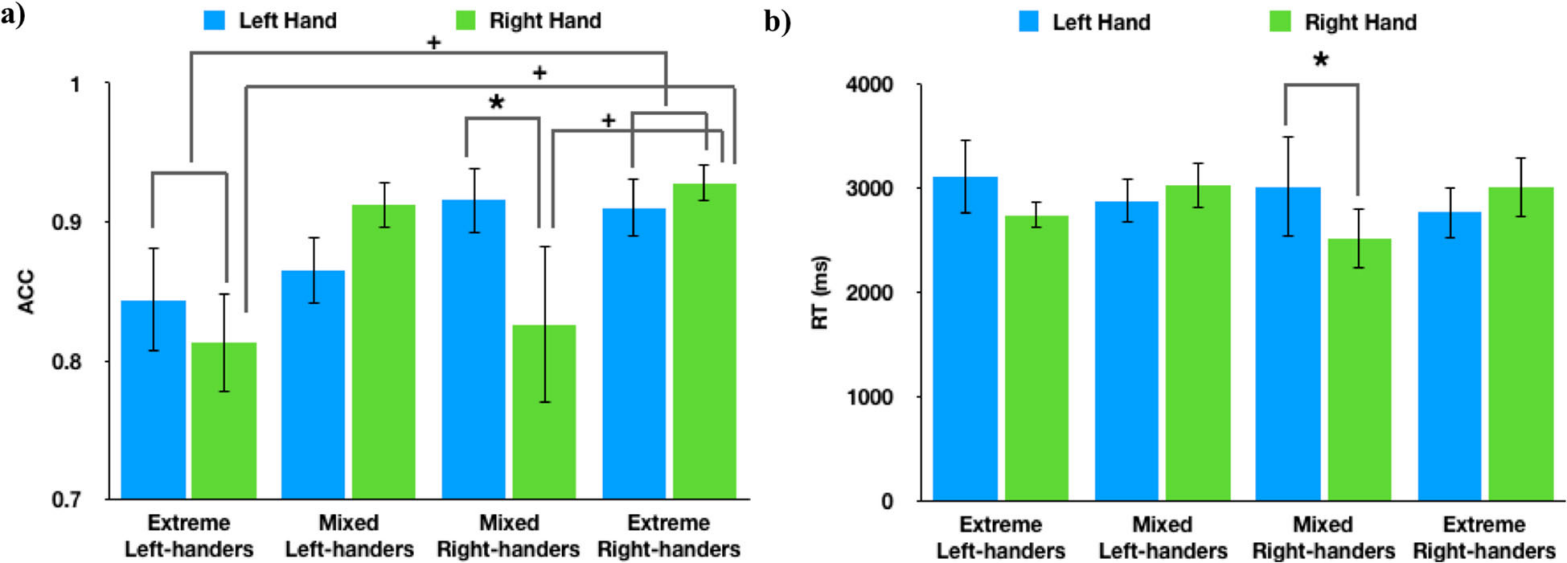
Performance for same-hand stimuli/same-gesture trials. Significance levels labeled in the figure are based on the handedness direction × handedness strength × hand stimuli tested three-way analysis of variance among all subjects. **a** Accuracy for same-hand stimuli/same-gesture trials. Mixed right-handers had significantly higher accuracy for left-hand stimuli than for right-hand stimuli (supports Hypothesis 3 or 4). Extreme right-hander’s accuracy was marginally higher than extreme left-handers (does not support any hypothesis). Extreme right-handers had marginally higher accuracy than mixed right-handers and extreme left-handers for right-hand stimuli (both support Hypothesis 2). In addition, mixed left-handers had a trend for higher accuracy for right-hand stimuli than for left-hand stimuli (supports Hypothesis 1 or 4). Mixed left-handers also had a trend for higher accuracy for right-hand stimuli than mixed right-handers (supports Hypothesis 4). **b** Reaction time for same-hand stimuli/same-gesture trials. Mixed right-handers responded faster for right hands than for left hands (supports Hypothesis 1 or 2). *ACC* Accuracy; *RT* Reaction time; ^+^*p* < .1; * *p* < .05

We conducted a 2 (handedness direction: left-handed, right-handed) × 2 (handedness strength: extreme, mixed) × 2 (hand stimuli tested: left hands, right hands) ANOVA on trials where the hand stimuli on the screen were both left hands or both right hands, and they were both making the same gesture^1^ (“same hand/same gesture”; see Table 3 and Fig. 5). For accuracy, there was a significant interaction between handedness direction and handedness strength [*F*(1,62) = 4.99; *p* = .03; *η*_*p*_^*2*^ = 0.07], but no other significant main effects or two-way interactions (all *p* > .1). Tukey *post hoc* tests revealed that extreme right-handers (*M* = 92% ± 1%) had marginally higher accuracy than extreme left-handers (*M* = 83% ± 3%; *p* = .084) for same hand/same gesture trials, but no difference was found between mixed left-handers (*M* = 89% ± 1%) and mixed right-handers (*M* = 87% ± 3%; *p* = .941). Finally, we found a significant three-way interaction among handedness direction, hand stimuli tested, and handedness strength [*F*(1, 62) = 6.43; *p* = .01; *η*_*p*_^*2*^ = 0.09].

**Table 3:** Descriptive statistics for accuracy of same-hand/same-gesture trials

Condition	Left-handed				Right-handed			
	Mixed		Extreme		Mixed		Extreme	
	*M*	*SD*	*M*	*SD*	*M*	*SD*	*M*	*SD*
Left-hand stimuli	86%	11%	84%	12%	92%	9%	91%	9%
Right-hand stimuli	91%	8%	81%	11%	83%	22%	93%	6%

*Note*: *M* mean, *SD* standard deviation

Tukey *post hoc* tests for the three-way interaction revealed that mixed right-handers had higher accuracy for left-hand stimuli (*M* = 92% ± 2%) than for right-hand stimuli (*M* = 83% ± 6%; *p* = .014). This advantage for left/nondominant hand stimuli than for right/dominant hand stimuli partially supports the predictions of both Hypothesis 3 and Hypothesis 4; Hypothesis 3 predicts all people will perform better on left-hand stimuli, while Hypothesis 4 predicts better performance on left-hand stimuli for right-handers only. Further analysis between handedness groups’ performance found that extreme right-hander’s accuracy for right-hand stimuli (*M* = 93% ± 1%) was marginally higher than both mixed right-hander’s (*M* = 83% ± 6%; *p* = .07) and extreme left-hander’s accuracy for right-hand stimuli (*M* = 81% ± 4%; *p* = .075); both results support Hypothesis 2. These fine-grained results of handedness strength could explain the null results in the broader analyses, since the extreme-hander’s and mixed-hander’s results were averaged over hand stimuli tested.

For reaction time, there was a significant three-way interaction among handedness direction, hand stimuli tested, and handedness strength [*F*(1,62) = 8.34; *p* = .005, *η*_*p*_^*2*^ = 0.12]. There were no significant main effects or two-way interactions (all *p* > .1; see Table 4). Tukey *post hoc* tests revealed that mixed right-handers responded faster for right-hand stimuli (*M* = 2514 ± 289 ms) than for left-hand stimuli (*M* = 3012 ± 484 ms; *p* = .023), but extreme-handers had no such difference. None of the other contrasts were significant. Here, the advantage of mixed right-handers for right-hand stimuli supports Hypothesis 1 and Hypothesis 2.

**Table 4:** Descriptive statistics for reaction time of same-hand/same-gesture trials

Condition	Left-handed				Right-handed			
	Mixed		Extreme		Mixed		Extreme	
	*M*	*SD*	*M*	*SD*	*M*	*SD*	*M*	*SD*
Left-hand stimuli	2873	1027	3106	1114	3012	1934	2760	1024
Right-hand stimuli	3024	1037	2742	411	2514	1155	3010	1182

*Note*: *M* Mean, *SD* Standard deviation. Reaction time is in milliseconds (ms)

On the basis of these results, none of the hypotheses align with all the data, and as there were indications that the patterns were different for extreme- and mixed-handed groups, we decided to look at the groups separately for a fine-grained examination of the role of handedness.

#### Same hand/same gesture: mixed-handed group

Mixed right-handers’ higher accuracy for left-hand stimuli and shorter reaction time for right-hand stimuli is indicative of a speed–accuracy trade-off. This finding replicates the results of a previous study (Ní Choisdealbha et al., 2011), although that study used a HLT instead of the same/different task (SMT). Thus, the overall results from the same-hand stimuli/same-gesture trials do not strongly support any of the hypotheses. Considering that extreme right-handers had an overall better performance than extreme left-handers and that extreme left-handers had a smaller sample size than the other three handedness groups, the data pattern in the mixed-handed group could be overshadowed in the three-way ANOVA that included extreme-handed groups. Therefore, we extracted data just for the mixed-handed groups for a *handedness direction* × *hand stimuli tested* two-way ANOVA.

For accuracy, there were no main effects (all *p* > .1), but there was a significant interaction between the two factors [*F*(1,37) = 6.44; *p* = .02; *η*_*p*_^*2*^ = 0.15]. Although Tukey *post hoc* tests did not find significant differences, the data pattern still showed a trend for a “wrong-hand effect”: Mixed left-handers tended to have higher accuracy for right-hand stimuli (*M* = 91% ± 2%) than for left-hand stimuli (*M* = 86% ± 2%); mixed right-handers tended to have higher accuracy for left-hand stimuli (*M* = 92% ± 2%) than for right-hand stimuli (*M* = 83% ± 6%). These results all showed a trend that mixed-handed groups had an advantage for nondominant hand stimuli than for dominant hand stimuli, which supports Hypothesis 4.

For reaction time, there were no main effects (all *p* > .1). There was a significant interaction between the two factors [*F*(1,37) = 4.94; *p* = .03; *η*_*p*_^*2*^ = 0.12], but *post hoc* tests did not find any significant differences or an obvious trend. Thus, although there were no major results for accuracy or reaction time for the mixed-handed group, there was an interaction in accuracy that lent some support to Hypothesis 4.

#### Same hand/same gesture: extreme-handed group

Similarly, we extracted data for the extreme-handed groups for a *handedness direction* × *hand stimuli tested* two-way ANOVA. For accuracy, there were no main effects or interactions (all *p* > .1), except a main effect of handedness direction [*F*(1,25) = 8.85; *p* = .006; *η*_*p*_^*2*^ = 0.26], such that extreme right-handers (*M* = 92% ± 1%) had overall higher accuracy than extreme left-handers (*M* = 83% ± 3%). For reaction time, there were no main effects (all *p* > .1), but there was a marginally significant interaction between the two factors [*F*(1,25) = 3.94; *p* = .06; *η*_*p*_^*2*^ = 0.14], but we did not examine this marginal result further.

### Strategy use

**Fig. 6:**
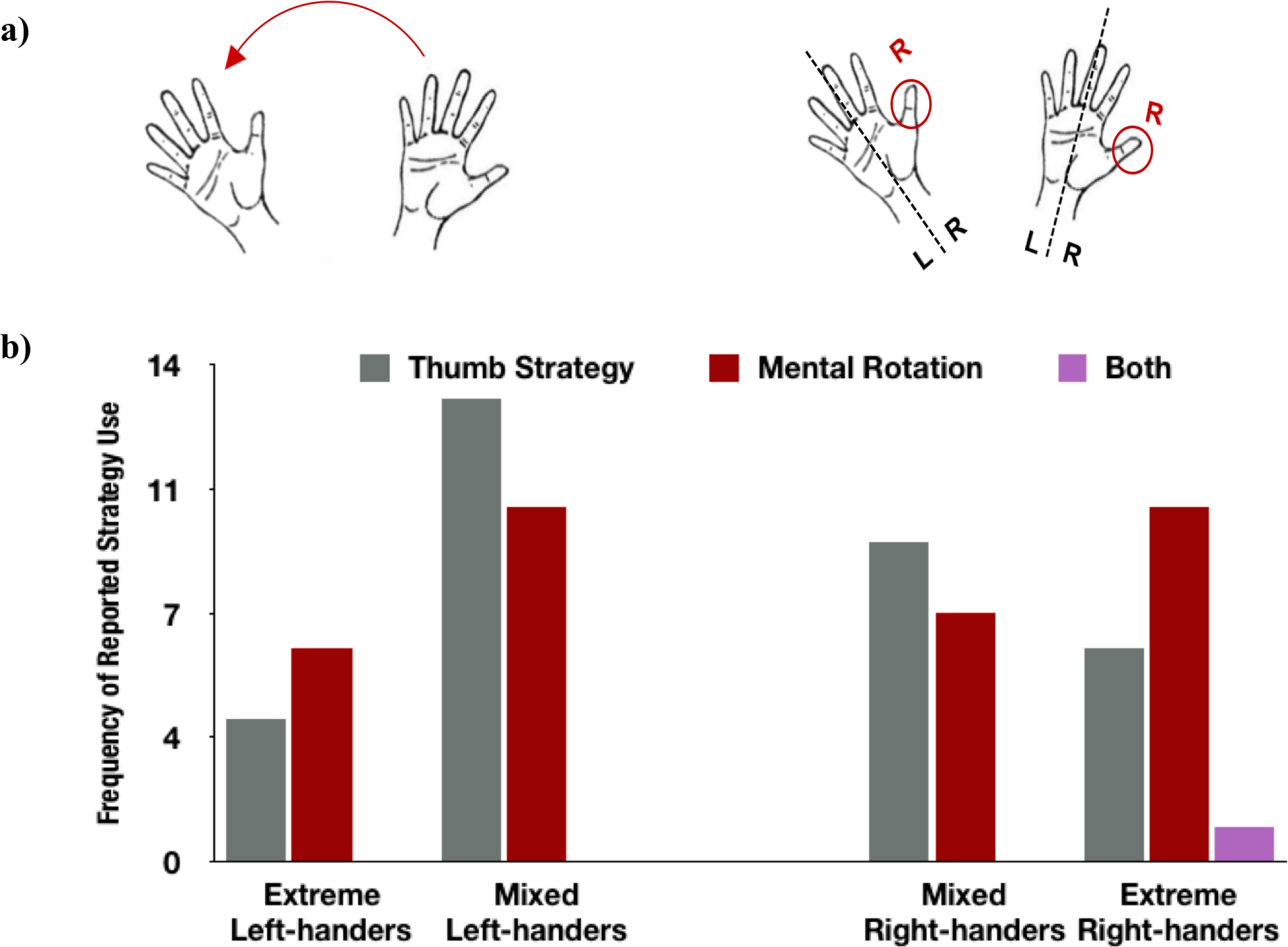
Mental rotation strategy. **a** Strategies used in the mental rotation of hands. The mental rotation strategy is to mentally rotate one hand to align it with the other hand. The thumb strategy is to compare the relative position of the thumb on each hand. *L* to the left of the hand central axis line; *R* to the right of the hand central axis line. **b** Frequency of reported strategy use. We found no group differences or interactions in strategy use

There were two main strategies reported in the hand MRT: the mental rotation strategy and the thumb strategy (see Methods and Fig. 6a). Both the mental rotation and thumb strategies were reported in all of the four handedness groups (extreme and mixed left- and right-handers; Fig. 6b). Each individual reported only one strategy, except one extreme right-hander who reported using both strategies.

Chi-square tests of independence revealed no significant difference among the four handedness groups on the two strategies (leaving out the subject who applied both strategies) [χ^2^(3, *N* = 65) = 2.026; *p* = .567, *ns*]. We also performed separate chi-square tests on the relationship between strategy use and handedness direction [χ^2^(1, *N* = 65) = 0.016; *p* = .9, *ns*] and handedness strength [χ^2^(1, *N* = 65) = 1.357; *p* = .244, *ns*], but no relationship was found. Further, we performed a 2 (handedness direction) × 2 (handedness strength) contingency log-linear analysis of subjects’ frequency of reported strategy use, with the two strategies (mental rotation, thumb strategy) as the contingencies. There were no main effects or interactions between handedness direction, handedness strength, and strategy (all *p* > .1). Although there was a higher proportion of subjects using the thumb strategy in the mixed-handed groups (56%) than in the extreme-handed groups (38%, not including the individual who applied both strategies), the difference was not statistically significant.

## Discussion

The goal of current study was to understand the mechanisms underlying the mental rotation of hands. We tested these mechanisms by applying a modified SMT with hand stimuli, testing left- and right-handed people. None of the hypotheses fully aligned with all the data; thus, we find that there is no single mechanism that underlies the mental rotation of hands. An overall analysis of same-hand trials revealed a marginal tendency for all participants to respond faster for right-hand stimuli than for left-hand stimuli, which supports world knowledge theory. For a nuanced examination of these mixed results, we took a fine-grained look into different groups. All groups performed similarly on left-hand stimuli; this consistent performance does not provide any support for embodied experience, whereas world knowledge might influence all groups similarly. In contrast, the variation in performance for right-hand stimuli across the groups was likely influenced by both world knowledge and embodied experience. Specifically, analysis within each handedness group demonstrated that mixed right-handers performed more accurately for left-hand stimuli, while mixed left-handers had a trend for higher accuracy for right-hand stimuli, supporting an embodied “wrong-hand effect.” In contrast, extreme right-handers had better performance than extreme left-handers overall. These findings suggest that these potential cognitive mechanisms might have differential effects on specific handedness groups.

### Comparison of hypotheses

Each of the four hypotheses is discussed below, along with the results. The results came from the analysis at the same-hand/same-gesture level if not specified.

Hypothesis 1 corresponds to the combination of the motor imagery and world knowledge theories. This hypothesis predicts that all subjects’ responses would be better for right-hand stimuli than for left-hand stimuli, and there would be no differences between the handedness groups. This prediction was supported by the overall analysis of all the same trials, such that all participants had a tendency to respond faster for right-hand stimuli than for left-hand stimuli, but the strength of this result is marginal. Additional support for Hypothesis 1 comes from the result that mixed right-handers responded faster for right-hand stimuli than for left-hand stimuli. However, this support is compromised due to a speed–accuracy trade-off, such that mixed right-handers had higher accuracy for left-hand stimuli than for right-hand stimuli, leading to no overall support of this theory. Some support for Hypothesis 1 comes from the lack of between-group differences for left-hand stimuli. However, this result was predicted by both Hypothesis 1 and Hypothesis 3 and is a null prediction, so it does not provide strong support.

Hypothesis 2 is the combination of the motor imagery and embodied experience theories. This hypothesis predicts that left-handers would respond better for left-hand stimuli than for right-hand stimuli and right-handers would respond better for right-hand stimuli than for left-hand stimuli. It also predicts that people with stronger right-hand strength would have better performance than people with weaker right-hand strength for right-hand stimuli and vice versa for left-hand strength. This prediction was supported by the result that extreme right-handers had marginally higher accuracy than both mixed right-handers and extreme left-handers for right-hand stimuli. However, this support is not very strong, because both results were marginally significant.

Hypothesis 3 is a combination of visual-proprioceptive integration and world knowledge theories. This hypothesis predicts that both left-handers and right-handers would respond better for left-hand stimuli than for right-hand stimuli, and there would be no differences between handedness groups either for left-hand stimuli or for right-hand stimuli. As mentioned before, we found that mixed right-handers had higher accuracy for left-hand stimuli than for right-hand stimuli, but this result was compromised by the speed–accuracy trade-off effect. Additionally, we found no between-group differences for left-hand stimuli, which provides weak support for this hypothesis.

Hypothesis 4 is a combination of visual-proprioceptive integration and embodied experience theories. This hypothesis predicts that left-handers would respond better for right-hand stimuli than for left-hand stimuli and that right-handers would respond better for left-hand stimuli than for right-hand stimuli. It also predicts that people with extreme right-hand strength would have worse performance for right-hand stimuli than people with mixed right-hand strength and vice versa for left-hand strength. Support for this hypothesis first comes from the result that mixed right-handers had higher accuracy for left-hand stimuli than for right-hand stimuli. The second result in support of this hypothesis is that mixed left-handers tended to have higher accuracy for right-hand stimuli than for left-hand stimuli. The third piece of support for this hypothesis is that mixed left-handers tended to have higher accuracy than mixed right-handers for right-hand stimuli. Although the evidence for Hypothesis 4 is fairly limited, including some marginal effects, it provides the strongest case across the four hypotheses.

In the analysis of all the same trials, we found that participants overall had a tendency to respond faster for right-hand stimuli than for left-hand stimuli. A previous study (Zapparoli et al., 2014) also found the right-versus-left reaction time advantage. However, there are a number of differences between their study and the present study. Their study used an HLT; it was conducted only on right-handed people; and the reaction time advantage was only present for the back-of-the-hand view stimuli, not for the palm-view stimuli. First, the robust findings across both right- and left-handers across the two studies indicates that the right-versus-left reaction time advantage might not be influenced by handedness. In addition, the convergence of the back-of-the-hand view in the Zapparoli et al. study and palm view in our study indicates that the advantage might not be influenced by the stimulus view per se. The difference between the egocentric perspective of the HLT and the object-based transformation of the SMT used here might explain why Zapparoli et al. only found this effect in the back-of-the-hand view and not the palm view. However, we did not test the back-of-the-hand view in the present study and so cannot say for certain how these different perspectives might interact.

### Within extreme- and mixed-handed groups

Mixed-handed groups and extreme-handed groups showed quite different patterns of results (see Fig. 5), which indicates that each of the handedness strength groups might be supported by different mechanisms, as was discussed in previous studies (Lyle et al., 2008; Lyle & Orsborn, 2011; Propper et al., 2005). Therefore, it is worth discussing the results within each strength group separately to explore the possibility that each strength group recruited different mechanisms to complete the task.

#### Mixed-handed groups: embodied experience and visual-proprioceptive integration

Mixed left-handers’ trend for better performance on their nondominant hand and their trend for better performance than mixed right-handers for right-hand stimuli converge on Hypothesis 4, embodied experience, and visual-proprioceptive integration theories. Mixed right-handers’ conflicting within-group accuracy and reaction time results could have two possible interpretations. The first interpretation is to consider the conflicting results as a speed–accuracy trade-off, which does not really support any hypothesis. This speed–accuracy trade-off result between left- and right-handed groups in mental rotation of hands was also found in a previous study that used an egocentric perspective task, the HLT (Ní Choisdealbha et al., 2011). The similar result of a speed–accuracy trade-off in the previous egocentric perspective and the current object-based transformation task indicates that the influence of handedness underlying mental rotation of hands might be independent of frame of reference (egocentric vs. allocentric).

An alternative speculation is that accuracy and reaction time represent discrete processes in the MRT: Accuracy could be considered a primary indicator to distinguish between hypotheses of the mental rotation process. In this case, mixed right-handers’ higher accuracy for their nondominant hand stimuli than for their dominant hand stimuli adds support to Hypothesis 4, which is most strongly supported in the mixed-handed groups.

#### Extreme-handed groups

Extreme-handed groups showed a very different pattern of results from mixed-handed people. Extreme right-handers had overall higher accuracy than extreme left-handers averaged over both left-hand stimuli and right-hand stimuli. Although the analysis revealed that extreme right-handers had better performance for right-hand stimuli than extreme left-handers did, in support of Hypothesis 2, it could be due to the overall performance discrepancy between the two extreme-handed groups. Additionally, extreme left-handers had a small sample size (ten subjects), which decreased the power of the data analysis and the cogency of potential data interpretations regarding this group. Thus, none of our hypotheses can suitably explain the performance within the extreme-handed groups in this study.

### Between extreme- and mixed-handed groups

#### Left-hand stimuli: world knowledge

There was no performance discrepancy among any of the four handedness groups for left-hand stimuli. This result only fits the null prediction of Hypothesis 1 and Hypothesis 3. More specifically, it indicates no embodiment effects, regardless of whether motor imagery or visual-proprioceptive integration is the underlying mechanism. World knowledge might instead be involved during the response to left-hand stimuli for all people, although this support is weak.

#### Right-hand stimuli: world knowledge and embodied experience

Despite homogeneous performance for left-hand stimuli, there were inconsistent results for right-hand stimuli between the extreme-handed group and the mixed-handed group. If embodied experience was the only factor in the response to right-hand stimuli, regardless of motor imagery or visual proprioceptive integration, then performance should increase in a set order across groups: extreme left-handers, mixed left-handers, mixed right-handers, extreme right-handers. If there is an additional influence of world knowledge for right-hand stimuli, this factor is assumed to influence all handedness groups similarly. Therefore, the predicted order in performance should stay the same. However, the “disrupted” order of the present results for right-hand stimuli (ranked from worst performance to best: extreme left-handers, mixed right-handers, mixed left-handers, extreme right-handers) suggests an additional influence of world knowledge that varies among the handedness groups.

The speculation that world knowledge impacts each handedness group differently has some empirical evidence derived from animal studies. In 1975, scientists created a left-handed world for right-handed mice and a right-handed world for left-handed mice (Collins, 1975). Their results support the hypothesis that handedness can adapt to the predominant cues in the world. In the left-handed world, some right-handed mice adapted to the world and became left-handers, while the remaining right-handed mice continued to use their right hand. An analogous adaptation occurred to those left-handed mice in the right-handed world: some left-handers turned into right-handers, while the remaining left-handed mice continued to use their left hand for food. This adaptation provides a model for mixed-handed groups in human studies, especially in explaining the conflicting results between the mixed-handed group and the extreme-handed group in the current study.

### Limitations and further research

Although the current study ended with somewhat mixed results, some possible follow-up studies might give a clearer answer to the research question. One solution is to test only extreme-handed individuals, since they might be less susceptible to adapting to world knowledge. Another solution is to emphasize right-hand stimuli in a modified paradigm, because no between-group differences were found for left-hand stimuli. Additional trials for right-hand stimuli could bring more power to the results. Given the number of contrasts, a larger sample size for both trials and participants would increase the power to detect effects. We also used both palm and pointer stimuli, which increased the difficulty, but palm gestures and pointer gestures had similar results. Future work might only include palm stimuli. We could also change how people make their responses, since using hands to respond could interfere with potential motor systems involved in mental rotation. A few subjects reported that pressing “S” with their right hand went against their typical experience, so using verbal responses could help. To fully test the wrong-hand effect, we could also include the back side of hands as stimuli, rather than just palms. In addition, including a wider spectrum of angular disparities would help to thoroughly consider the orientation of the stimulus. Finally, although we did not find sex differences in this study, the power to detect potential effects was limited by the sample size.

Future work could also make improvements in how to define handedness. For example, a recent study indicates that the EHI alone is insufficient to reveal handedness discrepancies in performing mental rotation of cube figures because the questionnaire includes items referring to motor behaviors that subjects do not exercise regularly (e.g., striking a match) (Pietsch & Jansen, 2019). Therefore, we could include performance-based metrics of handedness, such as finger tapping (Liu, Forrester, & Whitall, 2006) and grip strength (Massy-Westropp, Gill, Taylor, Bohannon, & Hill, 2011) to capture a full picture of handedness.

Finally, the current study tests the influences of world knowledge and embodied experience only at the figural scale, which is “﻿small in scale relative to the body and external to the individual, and can be apprehended from a single viewpoint” (Hegarty, Montello, Richardson, Ishikawa, & Lovelace, 2006). Because of the small scale, the results of our study could be specific to this task. Thus, it is unknown whether world knowledge and embodied experience will contribute to cognition at other spatial scales (i.e. vista, environmental, and geographical scales). This question should be examined in the future.

## Conclusions

The influence of handedness on spatial abilities is a research field that has been relatively neglected. Many psychology studies only recruit right-handers, which only provides partial answers to many questions. The results of the current study indicate that, for mixed-handed people, embodied experience is important in the mental rotation of hands, and the information is likely processed via a visual-proprioceptive integration cognitive mechanism, or “wrong-hand effect.” However, for extreme-handed people, the results only showed that extreme right-handers had an overall better performance than extreme left-handers. Across handedness groups, there was no significant variation in performance for left-hand stimuli, with only right-hand stimuli producing significant variation. The findings suggest that world knowledge might independently influence performance for left-hand stimuli, while the performance for right-hand stimuli is influenced by a combination of world knowledge and embodied experience. More importantly, this study provides a new approach to compare the influence of embodied experience and world knowledge in spatial tasks.

## Data Availability

The datasets used and/or analyzed during the current study are available from the corresponding author on reasonable request.

## Appendix

### Edinburgh Handedness Inventory

Please indicate your preferences in the use of hands in the following activities by putting + in the appropriate column. Where the preference is so strong that you would never try to use the other hand unless absolutely forced to, put ++. If in any cases you are really indifferent, put + in both columns.

Some of the activities require both hands. In these cases, the part of the task, or object, for which hand preference is wanted is indicated in brackets.

Please try to answer all the questions, and only leave a blank if you have no experience at all with the object or task.

**Table:**

		Left	Right
1	Writing		
2	Drawing		
3	Throwing		
4	Scissors		
5	Toothbrush		
6	Knife (without fork)		
7	Spoon		
8	Broom (upper hand)		
9	Striking Match (match)		
10	Opening box (lid)		
i	Which foot do you prefer to kick with?		
ii	Which eye do you use when using only one?		

## Footnotes

1 We were also interested in comparing results from different gesture types (pointers vs. palms), but we did not have enough trials to conduct this analysis.
